# Isoflurane induces Art2‐Rsp5‐dependent endocytosis of Bap2 in yeast

**DOI:** 10.1002/2211-5463.13302

**Published:** 2021-09-29

**Authors:** Fumi Kozu, Kanae Shirahama‐Noda, Yasuhiro Araki, Shintaro Kira, Hitoshi Niwa, Takeshi Noda

**Affiliations:** ^1^ Center of Frontier Oral Science Graduate School of Dentistry Osaka University Suita Japan; ^2^ Department of dental anesthesiology Graduate School of Dentistry Osaka University Suita Japan

**Keywords:** anesthetic, arrestin, endocytosis, transporter, ubiquitin

## Abstract

Although general anesthesia is indispensable during modern surgical procedures, the mechanism by which inhalation anesthetics act on the synaptic membrane at the molecular and cellular level is largely unknown. In this study, we used yeast cells to examine the effect of isoflurane, an inhalation anesthetic, on membrane proteins. Bap2, an amino acid transporter localized on the plasma membrane, was endocytosed when yeast cells were treated with isoflurane. Depletion of *RSP5*, an E3 ligase, prevented this endocytosis and Bap2 was ubiquitinated in response to isoflurane, indicating an ubiquitin‐dependent process. Screening all the Rsp5 binding adaptors showed that Art2 plays a central role in this process. These results suggest that isoflurane affects Bap2 via an Art2‐Rsp5‐dependent ubiquitination system.

## Graphical abstract

Bap2, an amino acid transporter localized on the plasma membrane in yeast, was endocytosed when yeast cells were treated with isoflurane, general anesthesia. Depletion of *RSP5*, an E3 ligase, and Art2 (arrestin) prevented this endocytosis and Bap2. Thus, isoflurane affects Bap2 via an Art2‐Rsp5‐dependent ubiquitination system.

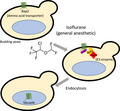

**Abbreviations**
Art2: arrestin‐related trafficking adaptors 2
Bap2: Branched‐chain amino acid permease 2
GFP: green fluorescent protein

General anesthesia is indispensable during surgical procedures in modern medicine. Anesthetics produce a variety of effects, such as loss of consciousness, immobilization, and suppression of the autonomic nervous system. It has been reported that anesthetics interfere with neurotransmission in many areas of the central nervous system, and the final site of action is most commonly thought to be the synaptic membrane. However, it remains unclear how anesthetics, particularly inhalation anesthetics, act on the synaptic membrane at the cellular level [1]. Current hypotheses regarding the mechanism of action of inhalation anesthetics can be classified as belonging to the membrane lipid theory or the membrane protein theory. The membrane lipid theory postulates that anesthetics act nonspecifically on lipids in neurons to change their membrane structure and induce anesthetic effects. However, it cannot be explained precisely why anesthesia occurs despite the existence of a huge number of target protein [2]. On the other hand, the membrane protein theory states that anesthetics produce their effects by directly binding to membrane proteins such as GABA_A_ receptors [3]. However, anesthetics with very different chemical structures produce the same general anesthetic effects, and it is quite hard to explain the various effects of anesthetics based on their actions on a single protein [2]. Thus, there are many issues that cannot be understood based on the membrane lipid theory or the membrane protein theory alone, and it is necessary to consider a new hypothesis.

Inhalation anesthetics have been reported to affect various cells and tissues, including mammalian neuronal and non‐neuronal cells, yeast cells, and bacteria [4, 5]. Keil *et al*. [4] reported that growth of the budding yeast *Saccharomyces cerevisiae* was inhibited by exposure to isoflurane, an inhalation anesthetic that is a halogenated ether with the chemical formula CF_3_CHCl‐O‐CF_3_. In general, isoflurane induces anesthesia in humans within 5 min [6]. Palmer *et al*. [7] also screened various *S. cerevisiae* mutants to determine which genes were involved in the effects of isoflurane. Their results showed that overexpression of the amino acid transporter Tat1 resulted in a 12% increment in resistance to isoflurane, which was further augmented by co‐overexpression of Bap2 [7]. In addition, Bap2 deletion increased the anesthetic sensitivity [7]. Tat1 and Bap2 are present on the cell membrane of yeast cells, and Tat1 takes up leucine, tryptophan, isoleucine, valine, and tyrosine [8, 9]. Bap2 is an amino acid transporter that takes up leucine, isoleucine, and valine [10]. In this study, we found that isoflurane treatment caused Bap2 and several other transporters on the cell membrane of budding yeast to be endocytosed and transported to the vacuole.

## Materials and methods

### Cell culture

Yeast media were YPD (1% Yeast Extract 2% peptone (BD Biosciences, Sparks, MD, USA), 2% glucose (Wako, Osaka, Japan)) or SCD (0.17% yeast nitrogen base without amino acids or ammonium sulfate (BD Biosciences), 0.5% ammonium sulfate (Nacalai Tesque, Kyoto, Japan), 0.5% bacto casamino acid (BD Biosciences), 20 µg·mL^−1^ tryptophan (Sigma‐Aldrich, St. Louis, MO, USA), 20 µg·mL^−1^ adenine (Sigma‐Aldrich), and 20 µg·mL^−1^ uracil (Sigma‐Aldrich), 2% glucose). Agar medium was supplemented with 2% agarose (Shoei Agar, Tokyo, Japan). For yeast culture, cells were precultured in YPD or SCD liquid medium at 30 °C and then inoculated into YPD or SCD liquid medium. After preculture at 30 °C, cells that reached the logarithmic growth phase (optical density at 600 nm (OD600) = 1 ˜ 2) were used for experiments.

Yeast cells were exposed to isoflurane (Wako) in a sealed environment comprising a 10‐mL syringe (Terumo, Tokyo, Japan, SS‐10SZP) or a 50‐mL syringe (SS‐50ESZ) covered by a cap (Top, Tokyo, Japan, JMDN70280000), in accordance with a previous report [11]. Yeast cells in the logarithmic growth phase (OD600=1 ˜ 2) were suspended in liquid medium, and the appropriate amount for each experiment was placed in a syringe. Isoflurane was diluted 10 times with DMSO (Wako), and the desired amount was added using a Hamilton syringe (Hamilton, NV, USA, 80465) with the tip of the syringe immersed in the liquid medium. The air was quickly removed, the cap was put on, and the plunger was pressed to confirm that it was sealed. The cells were then incubated at 30 °C for the appropriate duration for each experiment. Rapamycin (LKT Laboratories, St. Paul, MN, USA) was stored at 1 mg·mL^−1^ in stock solution (ethanol (Wako): Triton X‐100 (Wako) = 9 : 1 (v/v)) and added from the tip of the syringe to reach a final concentration of 200 ng·mL^−1^.

### Construction of yeast strains and plasmids

The yeast strains and plasmid types used in this study are listed in Tables 1 and 2, respectively. The following plasmids were used as templates for PCR fragment preparation. Gene disruptions are pFA6a‐natNT2, pFA6a‐kanMX6, and pFA6a‐zeoNT3. C‐terminal 3xHA tag added: pYM24. C‐terminal green fluorescent protein (GFP) tag added: pYM25. C‐terminal 3xGFP tag added: pFA6a‐3myeGFP‐natNT2. C‐terminal mCherry tag added: pKN12, pKN9. C‐terminal 5xFlag tag added: pFA6a‐5FLAG‐hphNT1. N‐terminal Tet07‐Ubi‐Leu‐3xHA tag added: pMK632. Primers were designed to produce a PCR fragment with a 20‐bp region homologous to the target DNA sequence at both ends, and PCR was performed using KOD Plus (Toyobo, Osaka, Japan). Parental strains in the logarithmic growth phase (OD=1 ˜ 2) were centrifuged at 1500 ***g*** for 2 min to collect the cells, which were then washed with sterile water. Cells were then soaked in 50 µL of 2 mg·mL^−1^ ssDNA (Sigma), 240 µL of 50% (w/v) polyethylene glycol (Sigma), and 36 µL of 1 m lithium acetate (Nacalai Tesque), and 25‐µL PCR fragments were added and vortexed. Transformation was carried out at 42 °C for 1 h. The generated strains were selected with clonNAT (Werner Bioagents, Jena, Germany) (100 µg·mL^−1^), G418 (Wako) (200 µg·mL^−1^), Zeocin (Life Technologies, Carlsbad, CA, USA) (200 µg·mL^−1^), or Hygromycin B (Wako) (200 µg·mL^−1^) added to the YPD medium. For PCR, colonies growing in the selection medium were suspended in zymolyase (Nacalai Tesque) solution (0.1 mg·mL^−1^) and incubated at 37 °C for 30 min prior to cell wall lysis and DNA was amplified using KOD FX (Toyobo).

**Table 1:** Yeast strains used in this study.

Strain	Genotype	Parent	References
BY4741	*MATa, his3D1 leu2D0 met15D0 ura3D0*		[35]
YMK119	*MATa, his3D1, leu2D0, met15D0, ura3D0, lys2::tTA, ura3::pCMVtetR'‐SSN6 KlURA3 (tetOFF)*	BY4741	[24]
SUB280	*MATa, lys2‐801, leu2‐3, ‐112, ura3–52, his3‐∆200, trp1–1, ubi1‐∆1::TRP1, ubi2‐∆2::ura3, ubi3‐∆ub‐2, ubi4‐∆2::LEU2 [pUB39] [pUB100]*	DBY1829	[25]
SUB592	*MATa, lys2‐801, leu2‐3, ‐112, ura3–52, his3‐∆200, trp1–1, ubi1‐∆1::TRP1, ubi2‐∆2::ura3, ubi3‐∆ub‐2, ubi4‐∆2::LEU2 [pUB39] [pUB100][pUB221]*	SUB280	[36]
FKY003	*BAP2‐yeGFP::hph*	BY4741	This study
FKY005	*LYP1‐yeGFP::hph*	BY4741	This study
FKY010	*BAP2‐3HA::hph*	BY4741	This study
FKY014	*∆end3::kan, BAP2‐yeGFP::hph*	FKY003	This study
MNY035	*SCH9‐6HA::hph*	BY4741	Laboratory stock
FKY015	*TET07‐UBI‐LEU‐3HA‐RSP5::nat*	YMK119	This study
FKY016	*BAP2‐yeGFP::hph, TET07‐UBI‐LEU‐3HA‐RSP5::nat*	FKY015	This study
FKY019	*∆npr2::nat, BAP2‐yeGFP::hph*	FKY003	This study
FKY021	*∆art1::kan, BAP2‐yeGFP::hph*	BY4741	This study
FKY022	*∆art2::kan, BAP2‐yeGFP::hph*	BY4741	This study
FKY023	*∆art3::kan, BAP2‐yeGFP::hph*	BY4741	This study
FKY024	*∆art4::kan, BAP2‐yeGFP::hph*	BY4741	This study
FKY025	*∆art5::kan, BAP2‐yeGFP::hph*	BY4741	This study
FKY026	*∆art6::kan, BAP2‐yeGFP::hph*	BY4741	This study
FKY027	*∆art7::kan, BAP2‐yeGFP::hph*	BY4741	This study
FKY028	*∆art8::kan, BAP2‐yeGFP::hph*	BY4741	This study
FKY029	*∆art9::kan, BAP2‐yeGFP::hph*	BY4741	This study
FKY030	*∆bsd2::kan, BAP2‐yeGFP::hph*	BY4741	This study
FKY031	*∆bul1::kan, BAP2‐yeGFP::hph*	BY4741	This study
FKY032	*∆bul2::kan, BAP2‐yeGFP::hph*	BY4741	This study
FKY033	*∆ear1::kan, BAP2‐yeGFP::hph*	BY4741	This study
FKY034	*∆rcr1::kan, BAP2‐yeGFP::hph*	BY4741	This study
FKY035	*∆rcr2::kan, BAP2‐yeGFP::hph*	BY4741	This study
FKY036	*∆sna3::kan, BAP2‐yeGFP::hph*	BY4741	This study
FKY037	*∆ssh4::kan, BAP2‐yeGFP::hph*	BY4741	This study
FKY038	*∆bul1::nat, ∆bul2::kan, BAP2‐yeGFP::hph*	FKY032	This study
FKY048	*∆vps4::nat, BAP2‐3HA::hph*	FKY010	This study
FKY055	*BAP2‐5FLAG::hph, CUP1‐6HIS‐MYC‐UBI::TRP1*	SUB592	This study
FKY056	*BAP2‐5FLAG::hph*	SUB592	This study

**Table 2:** Plasmids used in this study.

Plasmid	Description	References
pFur4‐GFP	pRS416/Fur4‐GFP	Laboratory stock
pGFP‐Hxt1	pRS416/GFP‐Hxt1	Laboratory stock
pDsRed‐Sec7	pRS316/DsRed‐Sec7	Laboratory stock
pFA6a‐natNT2	template, nat (KO)	[37]
pYM24	template, hph (3HA)	[37]
pYM25	template, hph (yeGFP)	[37]
pMK632	template, nat (Tet07‐Ubi‐Leu‐3HA)	[24]
pFA6a‐kanMX6	template, kan (KO)	[38]
pFA6a‐zeoNT3	template, zeo (KO)	[39]
pFA6a‐3GFP‐natNT2	template, nat (3yeGFP)	Laboratory stock
pKN12	template, nat (mCherry)	Laboratory stock
pKN9	template, hph (mCherry)	Laboratory stock
pFA6a‐5FLAG‐hphNT1	template, hph (5Flag)	Laboratory stock

### Microscopic observation

After yeast was cultured with a syringe, the liquid medium was centrifuged at 1500 ***g*** for 2 min and 1.5 µL was placed on a glass slide and covered with a cover glass. Live cells were observed using a DMI6000B epi‐fluorescence microscope (Leica Microsystems, Wetzlar, Germany). Images were processed using Adobe Photoshop CS4.

### Protein extraction

After yeast was cultured in syringes, the appropriate number of cells was collected and they were suspended in 100 µL of 0.2 m NaOH (Wako) and 1% 2‐mercaptoethanol (Wako) per OD, placed on ice for 10 min, and centrifuged at 17 800 ***g*** at 4 °C for 2 min. The supernatant was discarded, and 100 µL of 1× sample buffer (2% SDS (Nacalai Tesque), 100 mm DTT (Wako), 60 mm Tris/HCl (pH 6.8) (Sigma), 0.001% bromophenol blue (Sigma), 10% glycerol (Wako)) was added per 1 OD. The pellet was suspended by adding 100 µL per OD, heated at 100 °C for 5 min, and the centrifuged supernatant was used as the sample for SDS/PAGE.

### Analysis of phosphorylation

After incubation of yeast in a syringe, 10 OD of cells were collected and TCA (Wako) was added to achieve a final concentration of 6%. The solution was placed on ice for 5 min, followed by centrifugation at 9 100 ***g*** at 4 °C for 1 min. The supernatant was discarded, the cells were washed twice with acetone (Wako) stored at −20 °C, and the pellet was completely dried. The pellet was suspended in 100 µL urea buffer (50 mm Tris/HCl (pH 7.5), 5 mm EDTA (Wako), 6 m urea (Wako), 1% SDS, 1 mm PMSF (Wako), 0.5× Complete EDTA‐Free Protease Inhibitor Cocktail (Roche Life Science, Penzberg, Germany)), transferred to a screw‐cap tube, and incubated at 37 °C for 1 h. An equal volume of 0.6‐mm zirconia beads (Biomedical Sciences, Tokyo, Japan) was added to the pellet. Cells were disrupted at 5500 r.p.m. for 30 s using a bead‐type cell disruption device MS‐100 (Tommy Seiko, Tokyo, Japan), then placed on ice for 30 s. This process was repeated four times, and the cells were then centrifuged at 20 400 ***g*** at 4 °C for 10 min. The supernatant was transferred to a new tube, 15 µL of 1 m CHES (pH 10.5) (Wako) and 10 µL of 7.5 mm NTCB (Sigma) were added, and the tubes were stored overnight at room temperature. The remaining supernatant was used to quantify the amount of protein using a Protein Assay Bicinchoninate Kit (Nacalai Tesque). The next day, 25 µL of 4× sample buffer was added, and then, 1× sample buffer was added based on the results of protein quantification so that the concentration between samples was equal.

### Analysis of ubiquitination

After culturing yeast with a syringe, 50 OD of cells were collected, washed with sterile water, and suspended in 500 µL of buffer C (6 m guanidine‐HCl (Wako), 50 mm Na‐Phosphate (pH 8.0) (Wako), 10 mm Tris/HCl (pH 8.0), 300 mm NaCl (Wako), 5 mm NEM (Nacalai Tesque), 1 mm PMSF, 0.5× Complete EDTA‐Free Protease Inhibitor Cocktail (Roche Life Science)). Cells were transferred to a screw‐cap tube, and an equal volume of 0.6‐mm zirconia beads was added to the pellet. Then, the following procedure was repeated three times: cell disruption for 20 s using a FastPrep‐24 bead‐type cell disruption system (MP Biomedicals, Santa Ana, CA, USA), followed by placing the solution on ice for 2 min, and centrifugation at 20 400 ***g*** at 4 °C for 15 min. The same amount of supernatant was transferred to a new tube, and Ni‐NTA Agarose (Qiagen, Hilden, Germany) and imidazole (Wako) were added to obtain Buffer D at a final concentration 10 mm (8 m urea, 50 mm Na‐Phosphate (pH 8.0), 10 mm Tris/HCl (pH 8.0), 300 mm NaCl, 5 mm NEM, 1 mm PMSF, 0.5× Complete EDTA‐Free Protease Inhibitor Cocktail (Roche Life Science)). After washing four times, 60 µL of 2× sample buffer and 0.6 m imidazole were added and the solution was heated at 65 °C for 15 min. The centrifuged supernatant was used as the sample for SDS/PAGE.

### SDS‐PAGE, western blotting

In running buffer (25 mm Tris/HCl (pH 8.3), 191 mm glycine (Wako), 0.1% SDS), the supernatant of the sample was subjected to polyacrylamide gel electrophoresis to separate the proteins. Then, in a NA‐150 tank blotting apparatus (Nihon Eido, Tokyo, Japan), the proteins were separated using a PVDF membrane (GE Healthcare, Little Chalfont, UK) at 150 mA constant current for 70 min. Protein‐transferred PVDF membranes were incubated in PBS‐T (137 mm NaCl (Wako), 2.7 mm KCl (Sigma), 10 mm Na_2_HPO_4_·12H_2_O (Wako), 1.76 mm KH_2_PO_4_ (Wako), 0.1%) containing 1% skim milk (Morinaga Dairy, Tokyo, Japan). Blocking was performed using Tween‐20 (Wako) for 30 min at room temperature. Then, primary antibody diluted in PBS‐T containing 1% skim milk was reacted for 2 h at room temperature and washed three times with PBS‐T, and secondary antibody diluted in PBS‐T containing 1% skim milk was reacted for 45 min at room temperature. The antibody was then washed three times with PBS‐T and reacted with Luminata Forte Western HRP Substrate (Merck Millipore, Burington, MA, USA) or ECL Select (GE Healthcare) for 45 min at room temperature, and the bands were detected using a Versa Doc imaging system (Bio Rad, Hercules, CA, USA). Primary antibodies were anti‐HA 16B12 (Covance, Berkeley, CA, USA) 1/1000 dilution, anti‐Pgk (Invitrogen, Camarillo, CA, USA) 1/5000 dilution, and anti‐Flag M2 (Sigma) 1/3000 dilution, and secondary antibodies were HRP‐conjugated anti‐mouse IgG (SouthernBiotech, Birmingham, AL, USA) at 1/5000 dilution.

## Results

### Isoflurane treatment in the liquid phase inhibits yeast growth

**Fig. 1:**
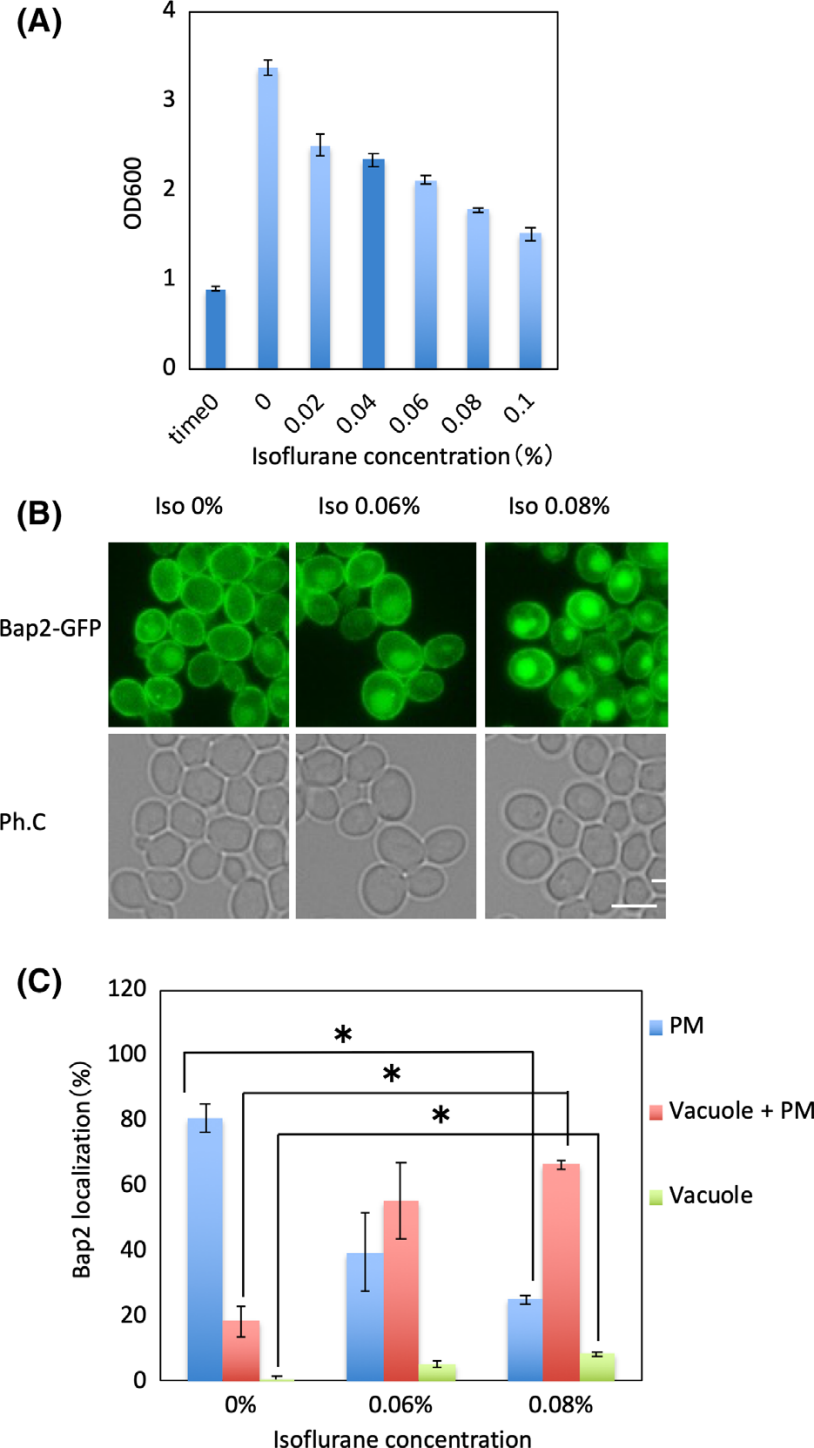
Isoflurane induces internalization of Bap2. (A) Wild‐type cells were grown in YPD medium. The culture was transferred to a syringe, and various concentrations of isoflurane were added. After 6‐h incubation, the optical density of each culture was analyzed. The average of three independent experiments and the standard deviation are shown. (B) Cells expressing Bap2‐GFP (FKY003) were grown in SCD. The culture was transferred to a syringe, and 0.06% or 0.08% isoflurane was added (shown as ‘Iso’). After 2‐h incubation, cells were analyzed by fluorescence microscopy. Bar, 5 µm. (C) Patterns of Bap2 localization (PM: plasma membrane, PM + Vacuole: plasma membrane and vacuole, Vacuole: vacuole only) are shown as percentages for each image. The average of three independent experiments (100 cells each) and the standard deviation are shown. *P < 0.05; by unpaired two‐tailed Student’s *t*‐test.

It was reported that the growth of yeast cells inoculated on plates was inhibited by 12% isoflurane in the gas phase [4]. This concentration is about 10 times higher than the minimum alveolar concentration of isoflurane (1.15%) in humans [12]. In the present study, treatment with isoflurane in the liquid phase was performed to approximate the environment in which cells are actually exposed to anesthetics in vivo. Although isoflurane is a liquid at room temperature, its high volatility makes it difficult to expose cells to a constant concentration using usual methods of liquid medium culture. Therefore, we optimized an experimental system for isoflurane treatment by using a syringe and cap in a sealed environment based on a previous study [11]. After 6 h of isoflurane treatment, yeast cell growth was suppressed in an isoflurane concentration‐dependent manner (Fig. 1A).

### Isoflurane affects the dynamics of various cell membrane transporters

It has been reported that yeast growth is suppressed by isoflurane treatment [4] and that this suppression is canceled by overexpression of amino acid transporters on the cell membrane [7]. In order to investigate the possibility that the localization of transporters is affected by isoflurane treatment, we labeled the transporters Bap2, Lyp1, Fur4, and Hxt1 with GFP. Bap2 localized to the plasma membrane in the absence of isoflurane, while isoflurane treatment resulted in typical vacuolar localization patterns in many cells (Fig. 1B). Treatment with 0.08% isoflurane resulted in vacuolar localization of Bap2 in about 75% of cells (Fig. 1C). Isoflurane treatment decreased the plasma membrane localization of the uracil transporter Fur4 and increased the vacuolar localization of the glucose transporter Hxt1 (Fig. S1A). However, the localization of the lysine transporter Lyp1 was not changed by isoflurane treatment (Fig. S1B). Thus, it is clear that isoflurane affects the dynamics of several transporters. We also observed Tat1‐GFP, and it was localized to the ER in response to isoflurane (data not shown). As this response was completely different from that of the other proteins examined, we decided to investigate Tat1‐GFP in future studies.

**Fig. 2:**
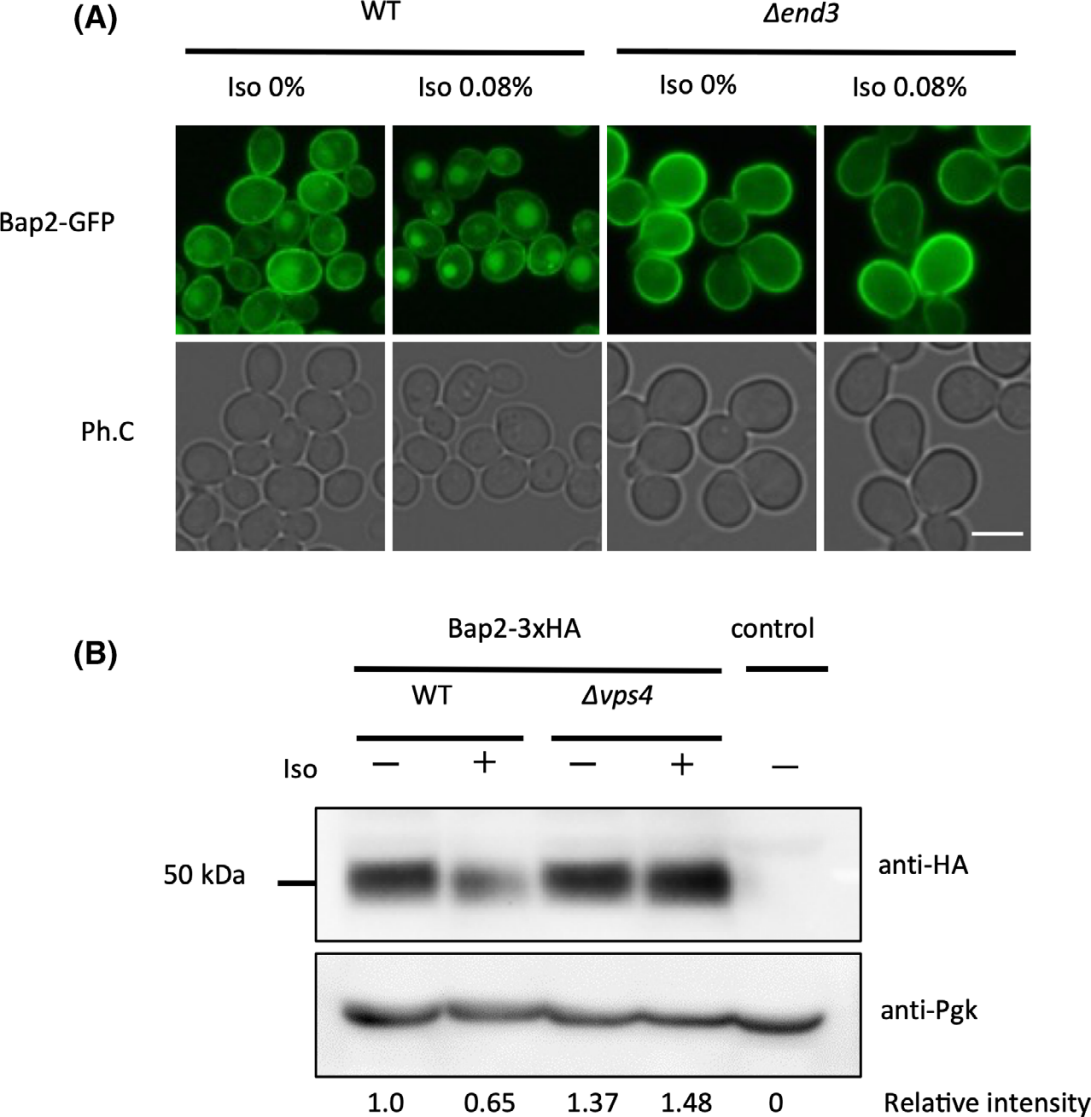
Isoflurane‐induced Bap2 relocation is dependent on endocytosis. (A) Cells of the indicated genotypes (wild‐type: FKY003, *∆end3*: FKY014) expressing Bap2‐GFP were grown in SCD. The culture was transferred to a syringe, and 0.08% isoflurane was added. After 2‐h incubation, cells were analyzed by fluorescence microscopy. Bar, 5 µm. (B) Cells of the indicated genotypes (wild‐type: FKY010, *∆vps4*: FKY048) expressing Bap2‐3xHA were grown in YPD. The culture was transferred to a syringe, and 0.08% isoflurane was added. After 2‐h incubation, lysates were analyzed by immunoblotting with anti‐HA antibody. The band intensity was measured using imagej (National Institute of Mental Health, Bethesda, MD, USA), and relative intensity adjusted with PGK was shown. ‘Control’ indicates BY4741 cells.

Normally, transporters on the plasma membrane are delivered to the vacuole via endocytosis, but there are also pathways in which transporters are newly synthesized and transported directly from the endoplasmic reticulum and Golgi apparatus to the vacuole without passing through the plasma membrane [13]. To determine whether the observed changes in transporter localization were caused by endocytosis, we generated a strain lacking End3, a protein involved in the formation of the actin skeleton in the early stage of endocytosis, and its deletion resulted in the loss of all endocytosis [14]. Bap2, which was localized to the vacuole by isoflurane treatment, was not transported to the vacuole in the *END3* deletion strain and instead remained localized at the plasma membrane (Fig. 2A). In the wild‐type strain, Bap2 was transported to the vacuole and then degraded, but the degradation was suppressed in the strain lacking Vps4, one of the ESCRT complexes involved in this transport (Fig. 2B) [15]. These results indicate that the vacuolar relocation of Bap2 by isoflurane treatment is due to its transport by endocytosis.

### Isoflurane‐Induced Endocytosis of Bap2 Involves a Different Regulatory Mechanism than TORC1

**Fig. 3:**
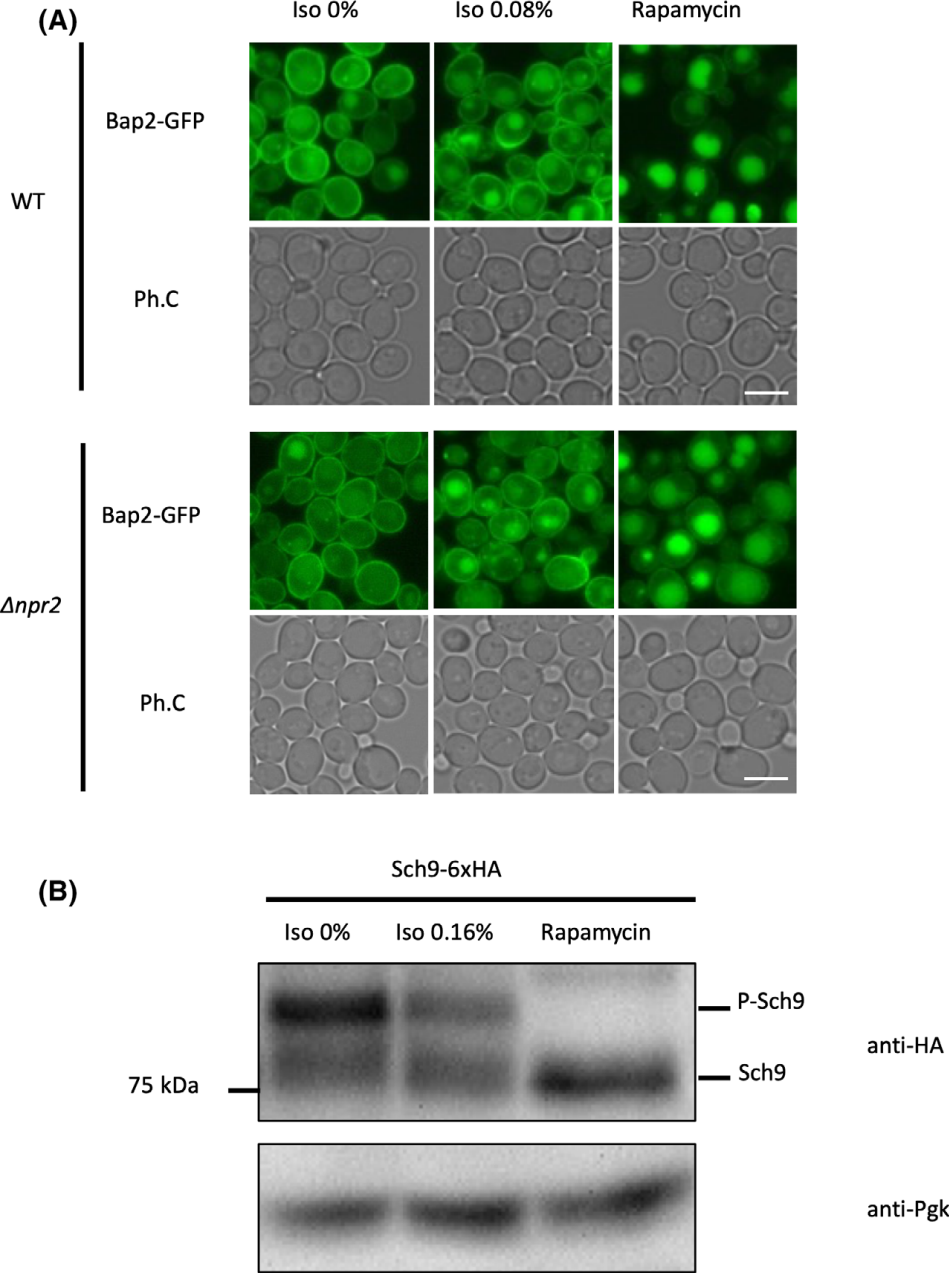
Endocytosis of Bap2 is independent of TORC1. (A) Cells expressing Bap2‐GFP (FKY003) and *∆npr2* (FKY019) were grown in SCD. The culture was transferred to a syringe, and 0.08% isoflurane or 200 ng·mL^−1^ rapamycin was added. After 2‐h incubation, cells were analyzed by fluorescence microscopy. Bar, 5 µm. (B) Cells expressing Sch9‐6xHA (MNY035) were grown in YPD. The culture was transferred to a syringe, and 0.16% isoflurane or 200 ng·mL^−1^ rapamycin was added. After 2‐h incubation, lysates were treated with NTCB and analyzed by immunoblotting with anti‐HA antibody.

TORC1 is a master regulator of cell proliferation that is widely conserved from yeast to mammals. It regulates translation, transcription, autophagy, and endocytosis of transporters [16]. Rapamycin suppresses TORC1, and it has been reported that rapamycin treatment promotes the degradation of Bap2 [17]. We observed the localization of Bap2 in the vacuole after rapamycin treatment, indicating promotion of endocytosis (Fig. 3A). Since isoflurane promotes endocytosis and degradation of Bap2, we investigated the possibility that isoflurane, like rapamycin, inhibits TORC1 and regulates these phenomena. Npr2 is a GTPase‐activating protein of Gtr1 that activates TORC1, and its deletion always activates TORC1 [18]. However, deletion of *NPR2* did not inhibit endocytosis of Bap2 by isoflurane treatment (Fig. 3A, *∆npr2*). Sch9, the direct substrate of TORC1, is phosphorylated and shows a band shift when TORC1 is activated, but it is dephosphorylated when TORC1 is inactivated by rapamycin treatment [17]. However, isoflurane treatment did not affect the phosphorylation status of Sch9 (Fig. 3B). These results suggest that isoflurane promotes the endocytosis of Bap2 through a mechanism other than TORC1.

### Isoflurane‐induced endocytosis of Bap2 is Rsp5 dependent

**Fig. 4:**
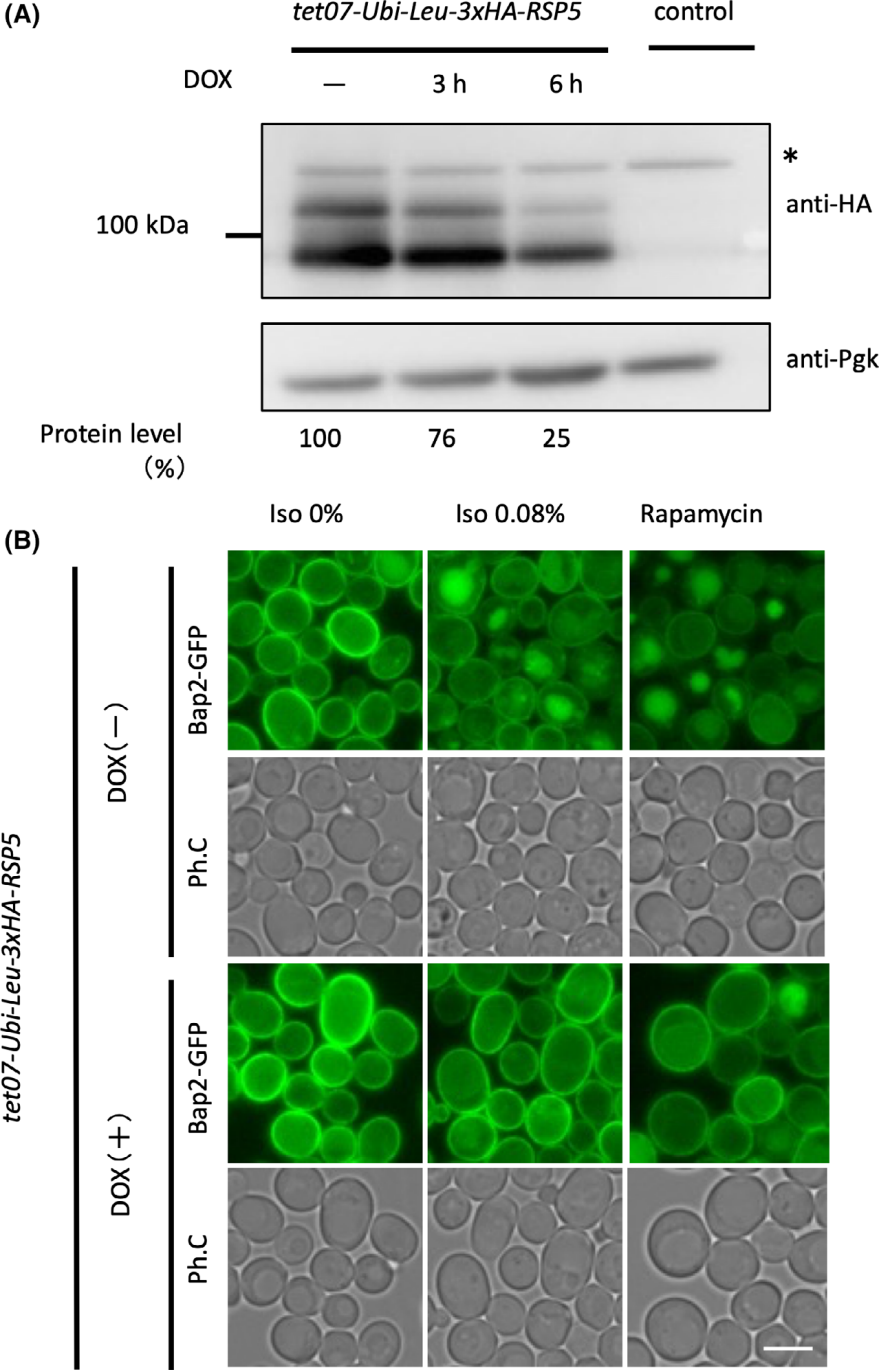
Isoflurane‐induced Bap2 endocytosis is inhibited in Rsp5‐depleted cells. (A) Cells expressing tet07‐Ubi‐Leu‐3xHA‐Rsp5 (FKY015) were grown in SCD medium containing 4 µg·mL^−1^ doxycycline (shown as ‘Dox’) for 3 or 6 h. Lysates were analyzed by immunoblotting with anti‐HA antibody. ‘Control’ indicates BY4741 cells. *: Nonspecific band. The protein level of Rsp5 is measured using imagej. (B) Cells expressing tet07‐Ubi‐Leu‐3xHA‐Rsp5 and Bap2‐GFP (FKY016) were grown in SCD medium containing 4 µg·mL^−1^ doxycycline for 6 h. The culture was transferred to a syringe, and 0.08% isoflurane or 200 ng·mL^−1^ rapamycin was added. After 2‐h incubation, cells were analyzed by fluorescence microscopy. Bar, 5 µm.

**Fig. 5:**
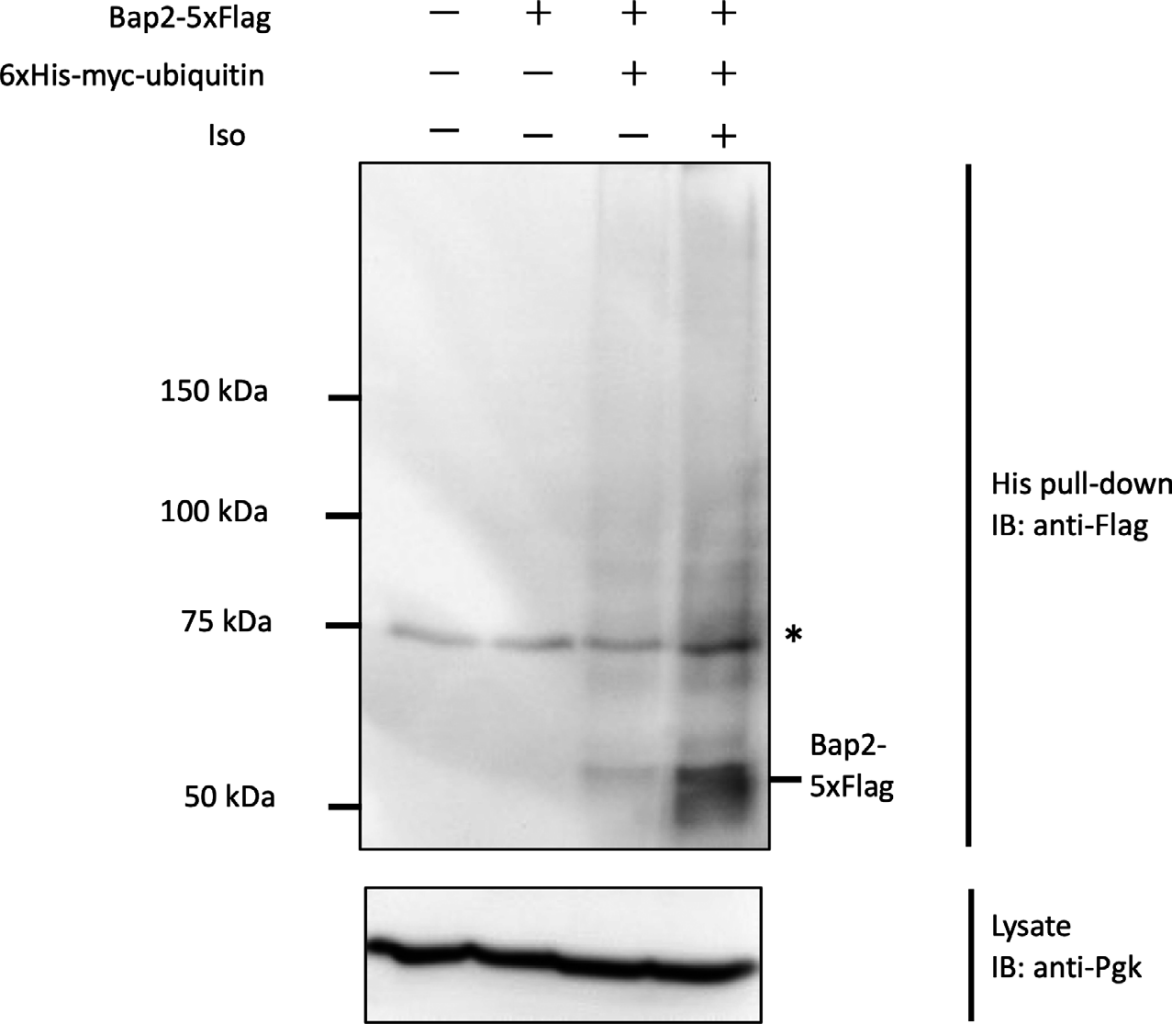
Isoflurane treatment induces ubiquitination of Bap2. Bap2‐5xFlag‐expressing cells, in which all ubiquitin genes were removed and a 6xHis‐myc‐ubiquitin‐coding plasmid was introduced (FKY055: lane 3, 4), were grown in YPD. The culture was transferred to a syringe, and 0.16% isoflurane was added. After 2‐h incubation, extracts were prepared and immunoprecipitated by Ni‐NTA beads. Precipitated proteins and whole‐cell extract (shown as ‘Lysate’) were analyzed by western blotting. As controls, cells that were similar to SUB592 except for either the introduction of a wild‐type ubiquitin plasmid (SUB280: lane 1) or the expression of Bap2‐5xFlag (FKY056: lane 2) were used. *: Nonspecific band.

Many transporters and receptor proteins on the cell membrane are quantitatively regulated by endocytosis, which is induced by ubiquitination in response to environmental changes such as nutrient starvation and cellular stress [19]. In mammalian cells, many types of ubiquitin ligases ubiquitinate various proteins on the plasma membrane, but in budding yeast, Rsp5, a HECT‐type ubiquitin ligase, is responsible for ubiquitination in most cases of endocytosis reported so far [20, 21, 22, 23]. Rsp5 is a member of the Nedd4 family and has nine homologs in humans, but it is the only Nedd4 family protein in budding yeast y. Since deletion of *RSP5* is lethal, we utilized doxycycline to generate a conditional knockdown strain of Rsp5 using the Tet‐off promoter, and destabilized the Rsp5 protein itself by changing the N terminus of Rsp5 to leucine to make it more susceptible to proteasomal degradation by the N‐terminal rule [24]. By culturing this strain for 6 h in the presence of doxycycline, the amount of endogenous Rsp5 protein could be reduced to about 25% of that before doxycycline treatment (Fig. 4A). When the cells cultured under these conditions were treated with isoflurane, Bap2 did not migrate to the vacuole but localized at the cell membrane even after isoflurane treatment (Fig. 4B). This indicates that the endocytosis of Bap2 is Rsp5 dependent. The same result was observed during rapamycin treatment, indicating that TORC1‐mediated endocytosis of Bap2 is also Rsp5‐dependent. The results of the ubiquitin pull‐down assay also suggested that isoflurane treatment resulted in the ubiquitination of Bap2 (Fig. 5). In this experimental system, the ubiquitin genes were deleted, a plasmid expressing 6xHis‐Myc‐Ubiquitin was inserted, and the target protein was tagged with 5xFlag [25]. The results showed that the Bap2‐5xFlag band was denser with isoflurane treatment than without, indicating that isoflurane treatment caused the ubiquitination of Bap2.

### Isoflurane‐induced endocytosis of Bap2 depends on Art2

**Fig. 6:**
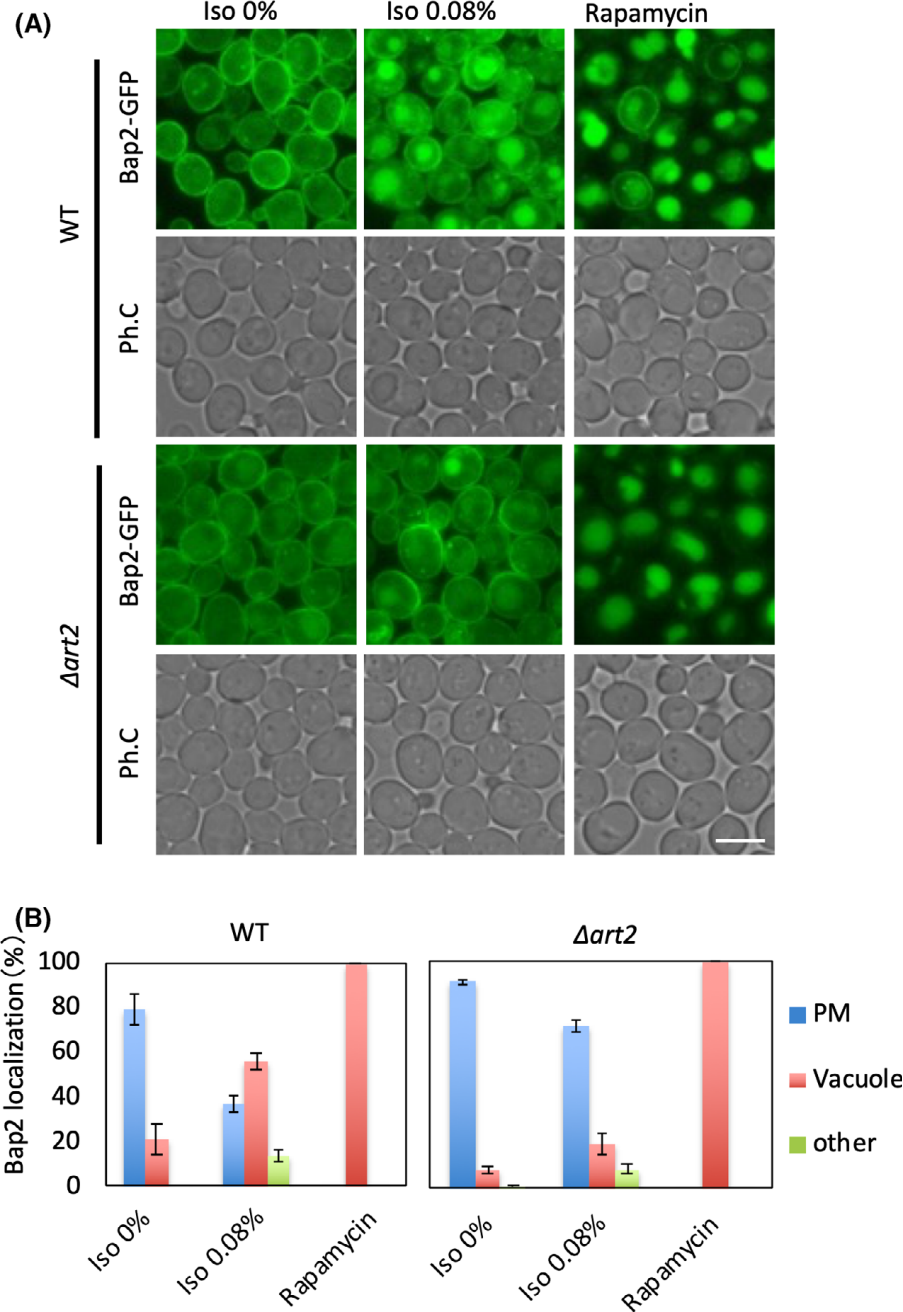
Isoflurane‐induced Bap2 endocytosis is inhibited in *∆art2* cells. (A) Cells of the indicated genotypes (wild‐type: FKY003, *∆art2*: FKY022) expressing Bap2‐GFP were grown in SCD. The culture was transferred to a syringe, and 0.08% isoflurane or 200 ng·mL^−1^ rapamycin was added. After 2‐h incubation, cells were analyzed by fluorescence microscopy. Bar, 5 µm. (B) Patterns of Bap2 localization (PM: plasma membrane, Vacuole: vacuole, other: other structures) are shown as percentages for each image. The average of three independent experiments (100 cells each) and the standard deviation are shown.

It has been reported that Rsp5 is recruited to each transporter by various adapter proteins when the transporters are ubiquitinated [26, 27]. The adaptor proteins contain PY motifs, through which they interact with the WW domain of Rsp5 [28]. There are more than a dozen types of adaptor proteins, and their involvement depends on the type of transporter they recognize and the nature of the environmental change [26, 27]. To identify the adaptor proteins involved in isoflurane treatment‐induced endocytosis of Bap2, we generated deletion strains of each of the 17 adaptor proteins with PY motifs and then assessed Bap2‐GFP localization after isoflurane treatment. The results showed that endocytosis of Bap2 was suppressed even after isoflurane treatment in the *ART2* deletion strain (Fig. 6A,B). Thus, we concluded that Art2 is involved in endocytosis of Bap2 in response to isoflurane.

## Discussion

To elucidate the mechanism of action of inhalation anesthetics in this study, we established an experimental system in which isoflurane was applied to yeast cells in the liquid phase, and showed that amino acid transporters on the cell membrane were endocytosed into vacuoles. It is known that numerous transporters, including Gap1, Can1, Tat2, and Smf1, are ubiquitinated in an Rsp5‐dependent manner and are endocytosed as a result of interaction between endocytosis executor molecules and ubiquitin recognition domains [29, 30, 31, 32]. In this study, we found that transient suppression of Rsp5 expression diminished Bap2 endocytosis, and Bap2 ubiquitination occurred during isoflurane treatment. Therefore, isoflurane‐induced endocytosis of Bap2 is also dependent on ubiquitination.

We also showed that Bap2 was endocytosed by inactivating TORC1. Since TORC1 activity was maintained during isoflurane treatment, it is likely that a mechanism other than TORC1 regulation was active during this time. The results of adapter protein screening showed that isoflurane promoted the endocytosis of Bap2 via Art2, and the accumulation of Art2 in the vicinity of the plasma membrane took about 30 min from the start of isoflurane treatment. Given these results, the mechanism of methionine transporter endocytosis is interesting [33]. In the absence of methionine, the methionine transporter Mup1 is localized at the plasma membrane, but in the presence of methionine, it is ubiquitinated by Art1‐Rsp5 and transported to the vacuole. In the presence of methionine, Art1‐Rsp5 is ubiquitinated and transported to the vacuole. The N‐terminal domain of Mup1, which is exposed to the cytoplasmic side, undergoes a conformational change in the presence of methionine, allowing Art1 to be recruited and to interact with it. Similarly, isoflurane treatment may induce a conformational change in Bap2 that is recognized by Art2. In future, it will be interesting to use the Art2‐dependent endocytosis found in this study to investigate how isoflurane alters the secondary and tertiary structures of Bap2. Interesting observations were recently reported on the mechanism of general anesthesia [34]. Super‐resolution microscopic observation revealed that treatment with inhaled anesthetics, including isoflurane, disrupts lipid rafts containing phospholipase D, thus leading to inactivation of K^+^ channels [34]. This constitutes the modified membrane lipid theory, and our results may be explained by disorganization of the membrane lipid domain. Although we only assessed the effects of isoflurane in this study, it is very possible that other inhalation anesthetics may have similar effects. A broader systematic analysis may reveal that inhalation anesthetics in general affect membrane proteins, which will hopefully lead to a deeper understanding of the underlying mechanism of these agents.

## Conflict of interest

The authors declare no conflict of interest.

## Author contributions

FK and TN conceived and designed the project; FK and KSN acquired the data; YA, SK, and HN analyzed and interpreted the data; and FK and TN wrote the study.

## Supporting information


**Fig. S1.** Isoflurane induces internalization of several plasma membrane transporters. A. Cells transformed with pFur4‐GFP or pGFP‐Hxt1 were grown in SCD. The culture was transferred to a syringe and 0.08% isoflurane or 200 ng/ml rapamycin was added. After 2‐h incubation, cells were analyzed by fluorescence microscopy. Bar, 5 µm. B. Cells expressing Lyp1‐GFP (FKY005) were grown in SCD, treated with 0.08% isoflurane, and analyzed by fluorescence microscopy as in A. Bar, 5 µm.

## Data Availability

The data that support the findings of this study are available from the corresponding author takenoda@dent.osaka-u.ac.jp upon reasonable request.
