# Supplementary figures and images for: Isoflurane induces Art2‐Rsp5‐dependent endocytosis of Bap2 in yeast
Source: FEBS Open Bio. 2021 Sep 29;11(11):3090–100. doi: 10.1002/2211-5463.13302 (PMC8564346; doi:10.1002/2211-5463.13302)

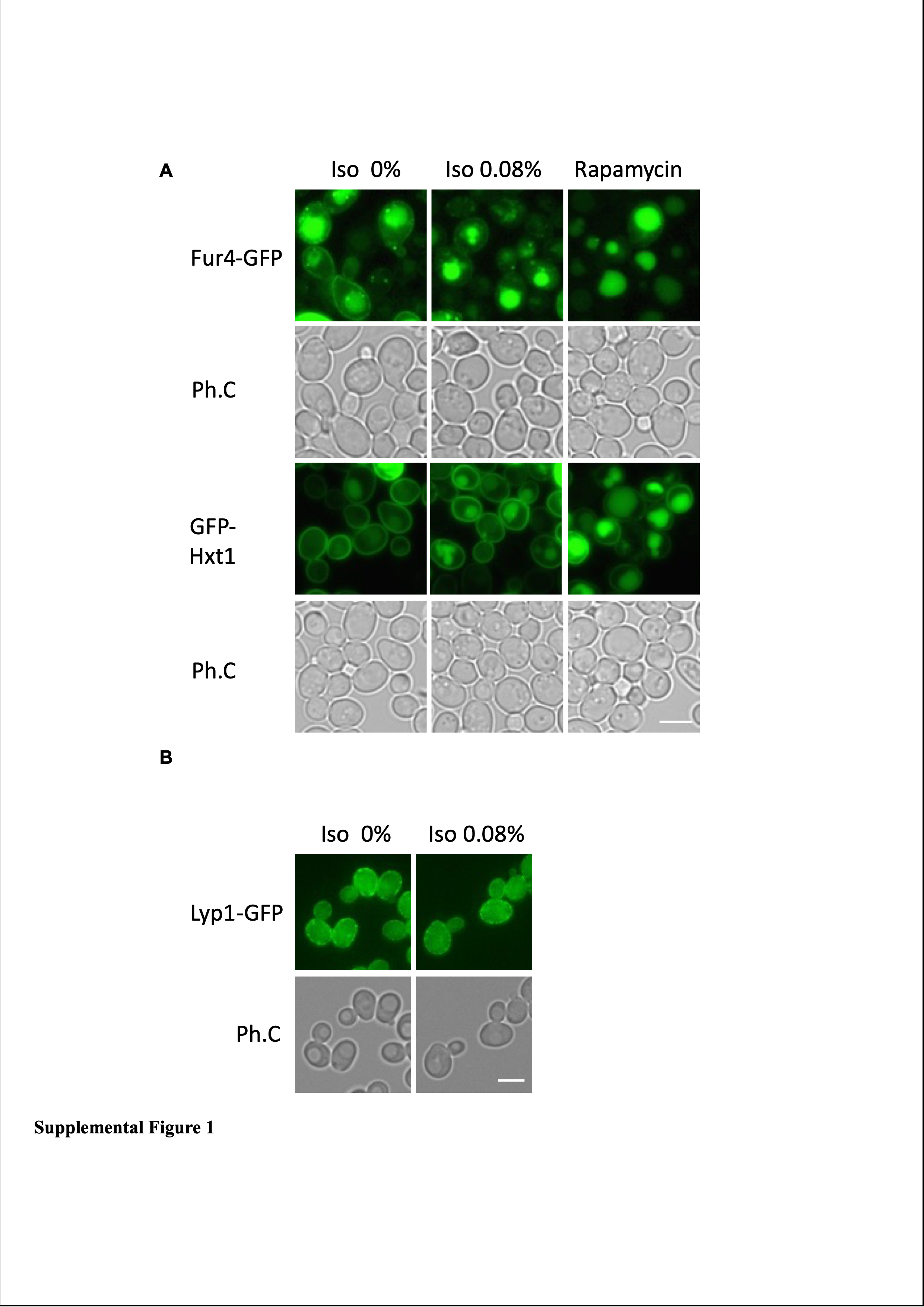

Supplement: Supplementary file 1 — Fig. S1. Isoflurane induces internalization of several plasma membrane transporters. A. Cells transformed with pFur4‐GFP or pGFP‐Hxt1 were grown in SCD. The culture was transferred to a syringe and 0.08% isoflurane or 200 ng/ml rapamycin was added. After 2‐h incubation, cells were analyzed by fluorescence microscopy. Bar, 5 µm. B. Cells expressing Lyp1‐GFP (FKY005) were grown in SCD, treated with 0.08% isoflurane, and analyzed by fluorescence microscopy as in A. Bar, 5 µm. [file FEB4-11-3090-s001.tiff]
